# Supplementary figures and images for: Early antiretroviral therapy for HIV-infected patients admitted to an intensive care unit (EARTH-ICU): A randomized clinical trial
Source: PLoS One. 2020 Sep 21;15(9):e0239452. doi: 10.1371/journal.pone.0239452 (PMC7505451; doi:10.1371/journal.pone.0239452)

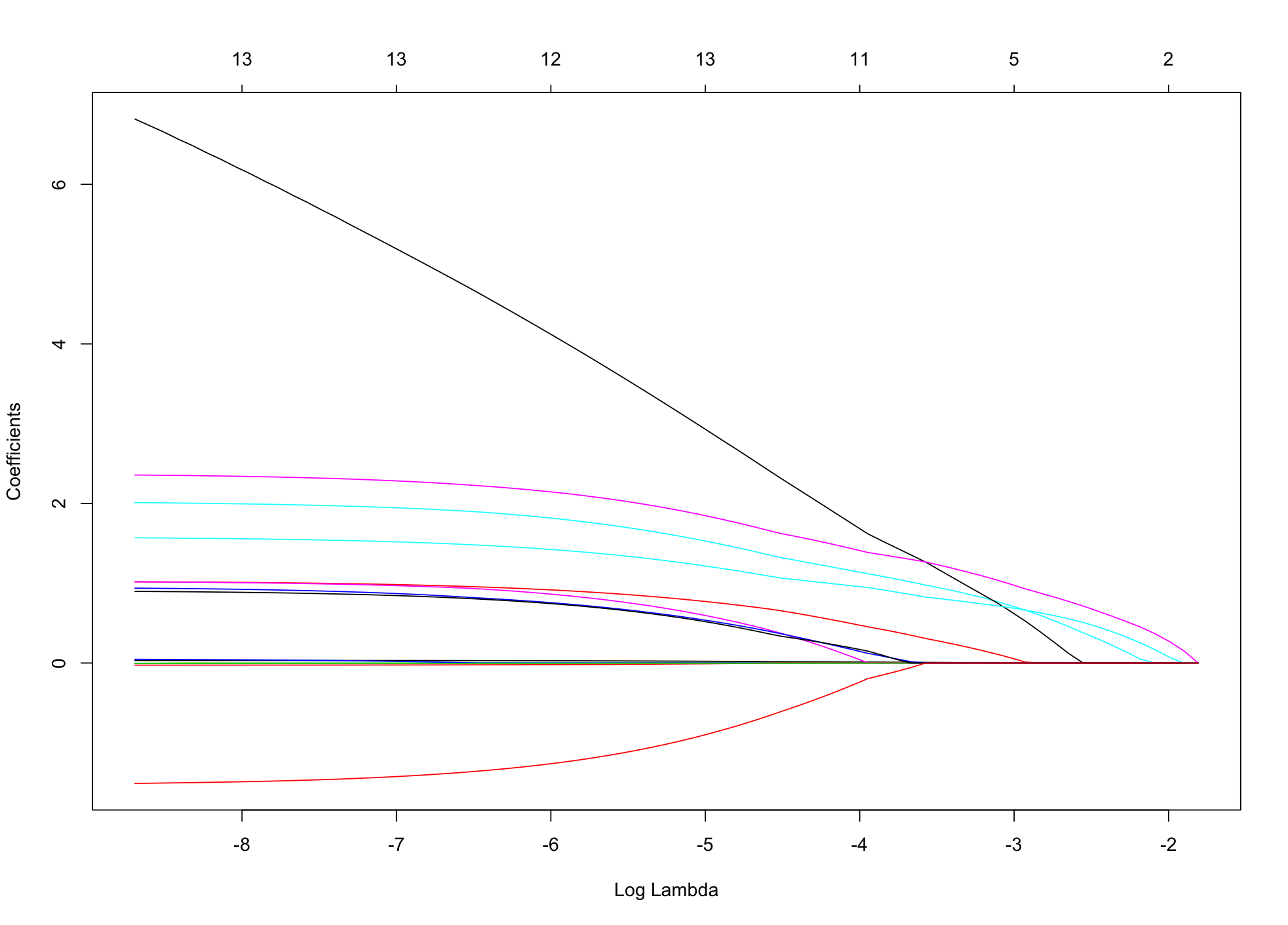

Supplement: S1 Fig — (PNG) [file pone.0239452.s002.png]

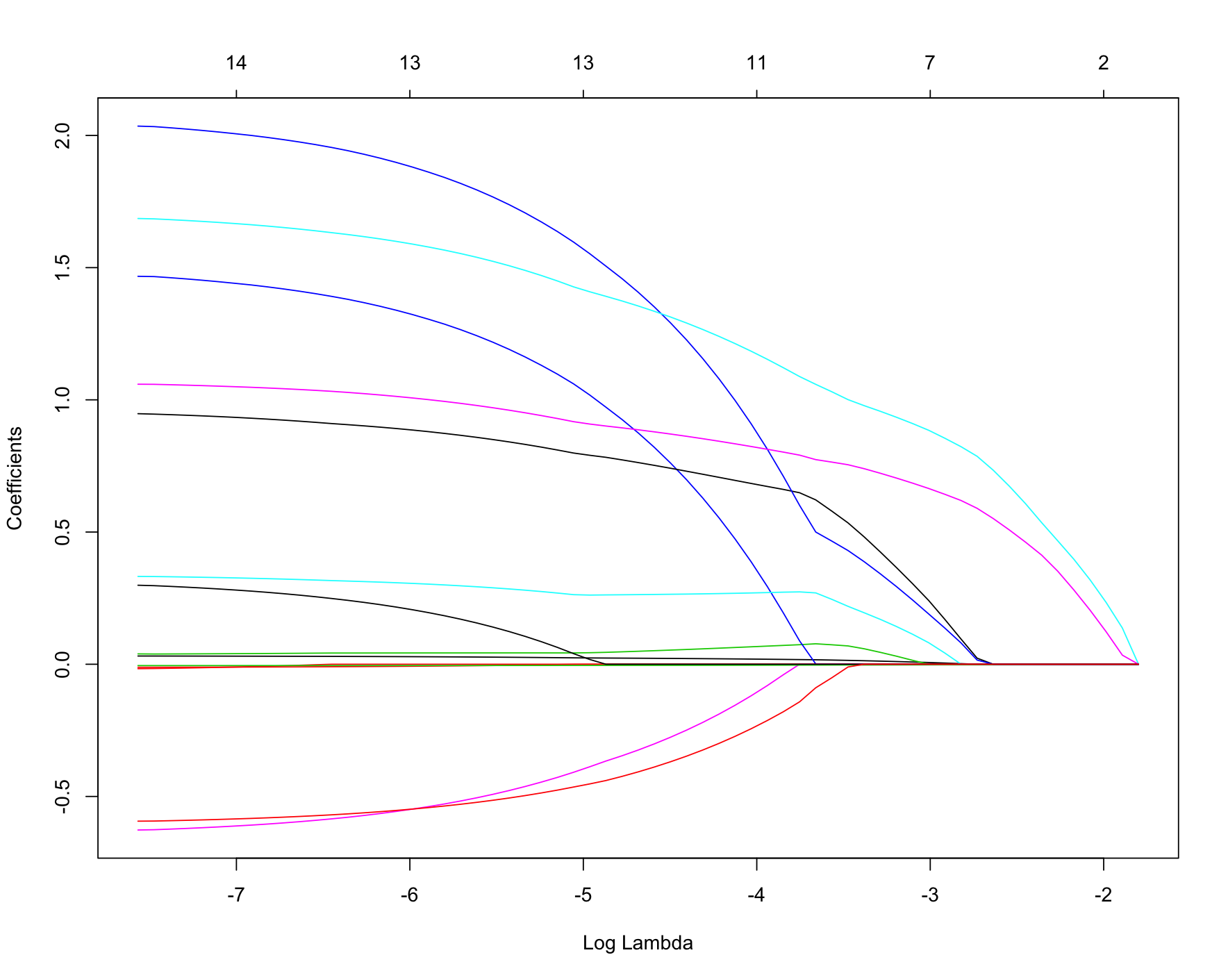

Supplement: S2 Fig — (PNG) [file pone.0239452.s003.png]

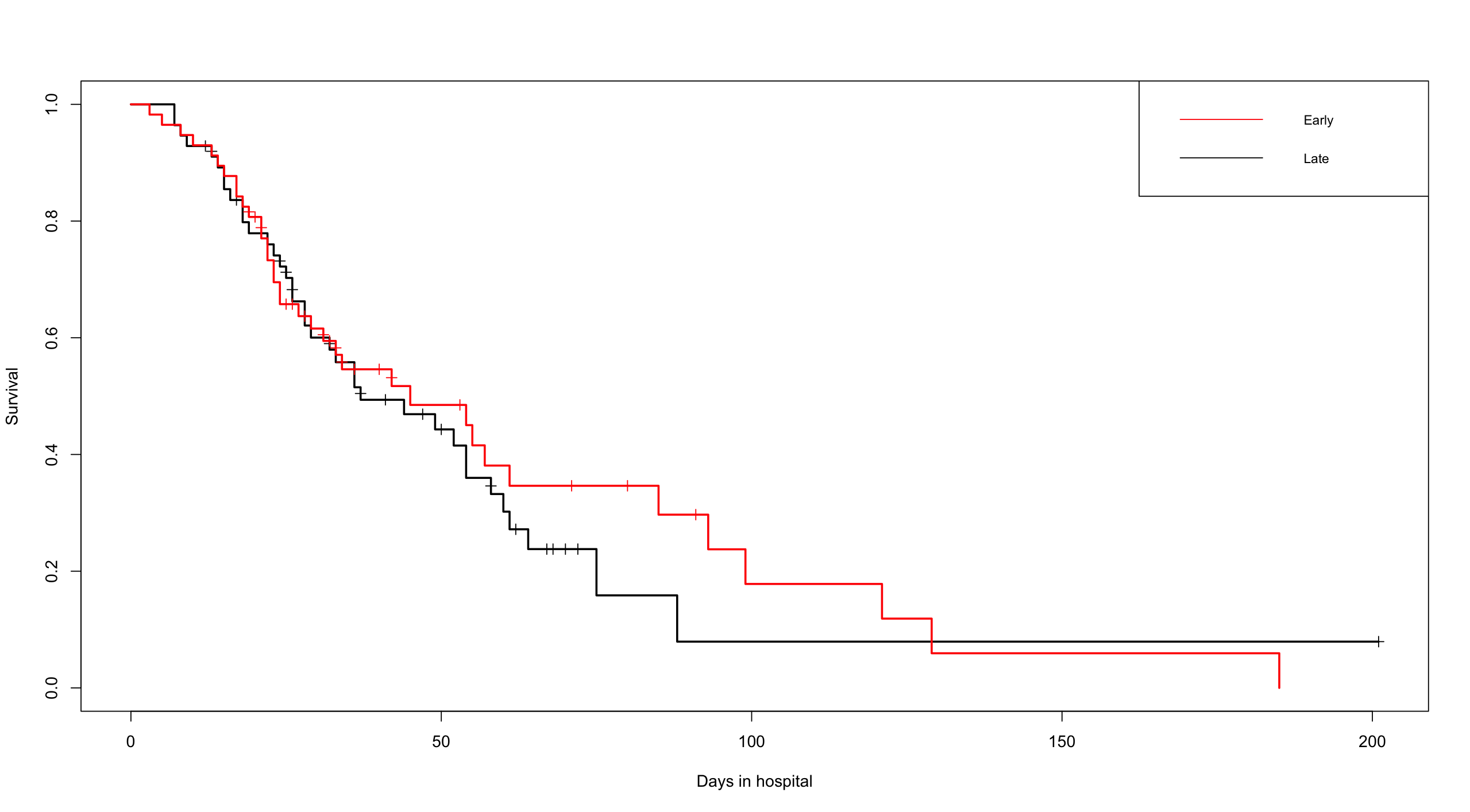

Supplement: S3 Fig — (PNG) [file pone.0239452.s004.png]
