# Supplementary material for: Early antiretroviral therapy for HIV-infected patients admitted to an intensive care unit (EARTH-ICU): A randomized clinical trial
Source: PLoS One. 2020 Sep 21;15(9):e0239452. doi: 10.1371/journal.pone.0239452 (PMC7505451; doi:10.1371/journal.pone.0239452)
Supplement: S1 File — (DOCX) [file pone.0239452.s005.docx]

1. Trial Protocol Study

**Early Antiretroviral Therapy in Critical HIV-Infected Patients**

Abstract

This study aims to evaluate the benefit of the early introduction of antiretrovirals (HAART) in patients infected with the Human Immunodeficiency Virus (HIV), hospitalized for acute illness in an Intensive Care Unit (ICU) environment. Design: Prospective, randomized, with two groups, one being early treatment (intervention) and another conventional treatment (control). Location: Medical-surgical intensive care unit of a tertiary hospital. Methods: Patients diagnosed with Acquired Immunodeficiency Syndrome (AIDS) who have been admitted to the ICU since January / 2012 will be included. The intervention group will receive HAART between the 2nd and 5th days of ICU stay and the control group will receive HAART after discharge from the ICU, according to the assistant team. The primary outcome to be analyzed will be hospital mortality. Secondary outcomes will be ICU and 6-month mortality.

INTRODUCTION AND LITERATURE REVIEW

Highly active and highly effective anti-retroviral therapy (HAART) has changed the natural history of HIV-infected patients. Before its advent, the disease behaved as potentially fatal. After the introduction of HAART, it started to acquire characteristics of chronic disease, with improved survival [1,2,3]. Despite this improvement, the number of ICU admissions for HIV-infected patients remained similar in the pre- and post-HAART periods [4], possibly due to the increase in the prevalence of chronic diseases in these patients [5,6]. Acute respiratory failure (ARF) is the most frequent syndromic diagnosis on admission to the ICU, being responsible for up to 42% of hospitalizations [7]. Among the causes of ARF, bacterial pneumonia is the main one, followed by Pneumocystis jirovecii pneumonia. Regarding the etiological diagnosis, more than half of admissions are related to non-opportunistic diseases, such as hepatitis C and hepatitis B virus co-infection, chronic obstructive pulmonary disease, renal failure, cirrhosis, cardiomyopathies and drug toxicity [8- 12].

The prognosis of HIV-infected patients admitted to the ICU in the short term is determined by the severity of the disease that motivated hospitalization [13,14]. Still, when comparing mortality in the ICU before and after HAART, no difference is detected [4,15]. Regarding long-term mortality, one study found that the two independent risk factors associated with mortality were the use of HAART (OR 0.45) and the diagnosis of AIDS on admission to the ICU (OR 2.46) ) [4].

Regarding the use of antiretroviral drugs, there is sufficient evidence to state that untreated HIV infection has negative health consequences at all stages of the disease [16]. In addition, new combinations of antiretroviral drugs are better tolerated than previous regimens, leading to greater treatment effectiveness and improved survival [16]. The North American recommendation, published in January 2011, developed by the Department of Health and Human Services (DHHS) defined that antiretroviral treatment should be initiated in all patients with a history of AIDS-defining disease or CD4 count <350 cells / mm3 [16]. Two randomized studies that compared HAART initiation with a CD4 cell count <350 cells / mm3 or <200 cells / mm3 showed a reduction in opportunistic infection and mortality in the group that started treatment earlier [17, 18]. In addition, there is an even earlier treatment trend (CD4 <500 cells / mm3) due to the evidence that HIV infection alone can contribute to a higher incidence and progression of cardiovascular, renal and liver diseases and neoplasias [19]. Regarding the beginning of treatment during ICU stay, studies are scarce. A retrospective study of patients admitted to the ICU for Pneumocistis jirovecci pneumonia showed a decrease in mortality among patients who were already using HAART or who started during hospitalization [20]. Croda et al, in a recent retrospective study, found that the use of HAART during hospitalization in the ICU was associated with increased survival in 6 months [21]. Other studies, however, have failed to show a difference in ICU and hospital mortality between patients with and without HAART [3,4]. In addition, the use of HAART in critically ill patients may be associated with serious side effects and increased resistance, the latter due to uncertain gastrointestinal absorption in these patients. Finally, the hepatic metabolism of many antiretroviral drugs can interfere with the metabolism of other medications administered in the ICU [22].

With the present study, we intend to assess whether the early onset of HAART in patients with HIV infection admitted to the ICU can be associated with reduced mortality.

Objectives

Primary objective: To verify whether there is a reduction in hospital mortality with early onset of HAART in HIV-infected patients admitted to the ICU.

Secondary objectives: To verify if there is a reduction in ICU mortality, 6-month mortality, and in length of stay in the ICU and in the hospital with early onset of HAART.

JUSTIFICATION

There are no prospective studies evaluating the use of HAART in the ICU. From the results of retrospective studies, a hypothesis was generated that this use could be associated with reduced mortality. This decision remains difficult, and is currently based on expert opinion.

The present study, due to its prospective design, intends to bring significant contributions regarding the impact of the use of HAART in the ICU, assessing the association with mortality, length of hospital stay and incidence of opportunistic infections, in addition to safety criteria.

METHODS

Location: Intensive Care Unit of Hospital Nossa Senhora da Conceição

Study Design:

Study type: Clinical trial

Allocation: Randomization

Blinding: not blinded

Population: Critically ill patients admitted to the ICU diagnosed with AIDS, without previous antiretroviral treatment, or without regular treatment in the last 3 months before admission.

Period: Beggining January 2012

Randomization: randomization will be done through a table of random numbers, in blocks and with previous stratification using the SAPS severity score 3 [23].

Sample size: Sample was calculated with the objective of reducing hospital mortality by 15% (with mortality in the control group 55%), with a significance level of 5% and study power 80%, requiring 172 patients in each group.

Study groups

- Early Intervention Group: HIV / AIDS patients, randomly allocated, to receive HAART between the 2nd - 5th days of ICU stay

- Conventional treatment group: HIV / AIDS patients, randomly allocated, to receive HAART after discharge from the ICU, at the discretion of the assistant team.

Inclusion criteria:

1. Older than 18 years old

2. HIV positive

3. a) CD4 + cell counts less than or equal to 350 cells / mm3 up to 3 months prior to study entry

or

 b) CD4 + cell count between 350 cells / mm3 and 500 cells / mm3 if older than 55 years, HCV (hepatitis C virus) or HBV (hepatitis B virus) co-infection, neoplasia, viral load> 100,000 copies / ml or high cardiovascular risk (Framingham score> 20).

or

c) AIDS-defining disease

Exclusion Criteria:

1. Regular treatment with HAART

2. Pregnancy

3. Tuberculous meningitis

4. Cryptococcal meningitis

5. Unable to use the gastrointestinal tract

6. ICU stay> 5 days before randomization

7. Refusal to participate in the study

Criteria for interrupting the intervention: Suspension of treatment due to side effects will be decided individually by the infectologist responsible for the case. Study interruption will be made if benefit is demonstrated in two consecutive analyzes with a difference of four standard deviations (p single-tail ≤ 0.00003) during the first half of the study and three standard deviations (p single-tail ≤ 0.002) in the second half.

Data collection: the data to be collected are described in the data collection form (Annex 1).

Statistical Analysis: Continuous variables will be presented as mean and standard deviation or median and interquartile range and compared with the Student's t-test or Mann-Whitney U test as indicated. The variables selected in the univariate analyzes (p <0.20) and the variables with biological plausibility will be subjected to multivariate analyzes by binary logistic regression. The results of the univariate and multivariate analyzes of logistic regressions will be expressed in odds ratios and respective 95% confidence intervals. In all analyzes, a level of p <0.05 will be adopted for statistical significance.

ETHICAL CONSIDERATIONS

The research will follow the recommendations of Resolution No. 196 of 10/10/96 - National Health Council (NHC) for Scientific Research in Human Beings. Researchers are committed to ensuring beneficence, committing themselves to maximum benefits and minimum damage and risks, and to non-maleficence, ensuring that predictable damage will be avoided. Our study will involve the introduction of antiretroviral medications in HIV-infected patients admitted to the ICU who have criteria for starting these drugs, based on the Brazilian Consensus on Antiretroviral Therapy of 2008 and the North American Recommendation of 2011 [16,24 ]. The study will obey the appropriate methodology, with random distribution of the research subjects in the groups of use of early intervention and conventional treatment. In addition, all participants or legal representatives must sign a consent form. We ensure that the proposed procedures will respect confidentiality and privacy, image protection and non-stigmatization, ensuring that information is not used to the detriment of study patients, including in terms of self-esteem, prestige and / or economic-financial . Concomitant to this, we guarantee the return of the benefits obtained through our research for people and the community through scientific publications. The data will be used only for this research, will be stored for a period of five years and, afterwards, destroyed.

BUDGET

The materials needed for the research, as well as their quantity, unit and total value are shown in the table.

There is no forecast of expenses with laboratory tests and / or medications. Laboratory tests will not be requested nor will medication be prescribed outside of daily clinical practice. Expected expenses will be for office supplies.

Table. Project budget

| **Material** | **Unit value (R$)** | **Quantity** | **Total value (R$)** |
| --- | --- | --- | --- |
| A4 sheets | 0.05 | 2000 | 100.00 |
| Printer cartridge | 40.00 | 1 | 40.00 |
| Pens | 2.50 | 4 | 10.00 |
| Total |  |  | 150.00 |

Own resources will be used, without burden to the Institution.

Schedule

|  | **2011** | **2012** | **2013** | **2014** | |
| --- | --- | --- | --- | --- | --- |
| **Activity** / M**onth** | Oct a Dec | Jan a Dec | Jan a Dec | Jan | Feb |
| Submission to Research Ethics Committee | **X** |  |  |  |  |
| Allocation of patients |  | **X** | **X** |  |  |
| Data analysis |  |  |  | **X** |  |
| Elaboration of manuscripts |  |  |  |  | **X** |

**REFERENCES**

1. Narasimhan M, Posner AJ, DePalo VA, Mayo PH, Rosen MJ. Intensive care in patients with HIV infection in the era of highly active antiretroviral therapy. *Chest*. 2004;125:1800-1804.

2. Rosen MJ. Intensive care of patients with human immunodeficiency virus infection: Time to take another look. *J Intensive Care Med*. 2005;20:312-313,314,315.

3. Dickson SJ, Batson S, Copas AJ, Edwards SG, Singer M, Miller RF. Survival of HIVinfected patients in the intensive care unit in the era of highly active antiretroviral therapy. *Thorax*. 2007;62:964-968.

4.Casalino E, Wolff M, Ravaud P, Choquet C, Bruneel F, Regnier B. Impact of HAART advent on admission patterns and survival in HIV-infected patients admitted to an intensive care unit. AIDS. 2004;18(10):1429-1433.

5. Morris A, Masur H, Huang L. Current issues in critical care of the human immunodeficiency virus-infected patient. Crit Care Med. 2006;34(1):42-49.

6. Vincent B, Timsit JF, Auburtin M, et al. Characteristics and outcomes of HIV-infected patients in the ICU: impact of the highly active antiretroviral treatment era. Intensive Care Med. 2004; 30(5):859-866.

7. Krista Powell, J. Lucian Davis, Alison M. Morris, Amy Chi, Matthew R. Survival for Patients With HIV Admitted to the ICU Continues to Improve in the Current Era of Combination Antiretroviral Therapy. *Chest* 2009;135;11-17

8. Nickas G, Wachter RM. Outcomes of intensive care for patients with human immunodeficiency virus infection. Arch Intern Med. 2000;160(4):541-547.

9. Rosen MJ, Clayton K, Schneider RF, et al. Intensive care of patients with HIV infection: utilization, critical illnesses, and outcomes. Pulmonary complications of HIV Infection Study Group. Am J Respir Crit Care Med. 1997;155(1):67-71.

10. Afessa B, Green B. Bacterial pneumonia in hospitalized patients with HIV infection: the pulmonary complications, ICU support, and prognostic factors of hospitalized patients with HIV (PIP) study. Chest. 2000;117(4):1017-1022.

11. De Palo VA, Millstein BH, Mayo PH, Salzman SH, Rosen MJ.Outcome of intensive care in patients with HIV infection. Chest.1995;107(2):506-510.

12. Narasimhan M, Posner AJ, DePalo VA, Mayo PH, Rosen MJ.Intensive care in patients with HIV infection in the era of highly active antiretroviral therapy. Chest. 2004;125(5): 1800-1804.

13. Casalino E, Mendoza-Sassi G, Wolff M, et al: Predictors of short-and long-term survival in HIV-infected patients admitted to the ICU.*Chest* 1998; 113:421–429

14. Bhagwanjee S, Muckart DJJ, Jeena PM, et al: Does HIV status influence the outcome of patients admitted to a surgical intensive care unit? A prospective double blind study. *BMJ* 1997; 314:1077–1084

15. Khouli H, Afrasiabi A, Shibli M, Hajal R, Barrett CR, Homel P. Outcome of critically ill human immunodeficiency virus-infectedpatients in the era of highly active antiretroviral therapy. J Intensive Care Med. 2005;20(6):327-333

16. Guidelines for the Use of Antiretroviral Agents in HIV-1-Infected Adults and Adolescents, [http://www.aidsinfo.nih.gov/ContentFiles/AdultandAdolescentGL.pdf](http://../../Library/Mail%20Downloads/%20http:/www.aidsinfo.nih.gov/ContentFiles/AdultandAdolescentGL.pdf), *janeiro, 2011*

17. Zolopa A, Andersen J, Powderly W, et al. Early antiretroviral therapy reduces AIDS progression/death in individuals with acute opportunistic infections: a multicenter randomized strategy trial. PLoS One. 2009;4(5):e5575

18. The SMART/INSIGHT and the D:A:D Study Groups TSIatDADSG. Use of nucleoside reverse transcriptase inhibitors and risk of myocardial infarction in HIV-infected patients. *AIDS.* 2008;22(14):F17-24.

19. Baker JV, Peng G, Rapkin J, et al. CD4+ count and risk of non-AIDS diseases following initial treatment for HIV infection. *AIDS.* 2008;22(7):841-848.

20. Morris A, Wachter RM, Luce J, Turner J, Huang L. Improved survival with highly active antiretroviral therapy in HIVinfectedpatients with severe Pneumocystis carinii pneumonia. AIDS. 2003;17(1):73-80.

21. Croda J, Croda MG, Neves A, De Sousa dos Santos S. Benefit ofantiretroviral therapy on survival of human immunodeficiency virus-infected patients admitted to an intensive care unit. Crit Care Med. 2009;37(5):1605-1611.

22. Huang L, Quartin A, Jones D, Havlir DV. Intensive care of patients with HIV infection. N Engl J Med. 2006;355(2):173-181.

23. Moreno RP, Metnitz PGH, Almeida E, et al: SAPS 3 - From evaluation of the patient to the evaluation of intensive care unit. Part 2: Development of a prognostic model for hospital mortality at ICU admission. *Intensive Care Med* 2005; 31:1345-1355

24. Ministério da Saúde. Recomendações para terapia anti-retroviral em adultos e adolescentes infectados pelo HIV 2007/2008. http://bvsms.saude.gov.br/bvs/ publicacoes/recomendacao_terapia.pdf

**Data collection form**

**ICU admission**

Code _____________________

Gender ( ) Male ( ) Female

Age ______

Procedência ( ) Emergency ( ) Ward ( ) Another hospital

ICU admission diagnosis___________________________________________

SAPS 3 _______

Admission date: Hospital ___/___/___ ICU ___/___/___

Length of hospitalization prior to the ICU (days) ___

Previous AIDS diagnosis ( )

Previous use of HAART ( ) Regular ( )

CD4+ count _____ Viral load _____________

Opportunistic infection on admission ( ) _________________________________

Co-infections: Hepatitis B ( ) Hepatitis C ( ) Syphilis ( ) Tuberculosis ( )

**Randomization**

Start of HAART in the ICU ( )

**After randomization**

AZT ( ) 3TC ( ) ddI ( ) d4T ( ) ABC ( ) TDF ( )

EFZ ( ) NVP ( )

LPV ( ) ATV ( ) IDV ( ) NFV ( ) RTV ( ) SQV ( )

APV ( ) ( )_________________________________________________

HAART start date ___/___/___ Days until HAART start ______

Delay at start ( ) __________________________________________________

Infections in the ICU ( ) ____________________________________

Opportunistic infections in the ICU ( ) _______________________________

Adverse effects ( ) _______________________________________________

Suspension ( ) ____________________ Exchange ( ) _____________________

Restart ( ) ___/___/___

Time on MV (days) ____

Date of discharge from ICU ___/___/__ Alive ( )

ICU LOS _____________

Date of discharge from hospital ___/___/___ Alive ( )

Hospital LOS _____

Six months after discharge - alive ( )
