# Supplementary material for: Early antiretroviral therapy for HIV-infected patients admitted to an intensive care unit (EARTH-ICU): A randomized clinical trial
Source: PLoS One. 2020 Sep 21;15(9):e0239452. doi: 10.1371/journal.pone.0239452 (PMC7505451; doi:10.1371/journal.pone.0239452)
Supplement: S2 File — (DOCX) [file pone.0239452.s006.docx]

1. Projeto de Pesquisa
2. **Terapia Anti-retroviral Precoce em Pacientes Críticos Infectados pelo HIV**

**RESUMO**

O presente estudo pretende avaliar o benefício da introdução precoce de anti-retrovirais (ARV) em pacientes infectados pelo vírus da Imunodeficiência Humana (HIV), internados por doença aguda em ambiente de Unidade de Terapia Intensiva (UTI). *Delineamento:* Estudo prospectivo, randomizado, com dois grupos, sendo um tratamento precoce (intervenção) e outro tratamento convencional (controle). *Local:* Unidade de tratamento intensivo clínico/cirúrgico de um hospital terciário. *Métodos*: Serão incluídos pacientes com diagnóstico de Síndrome da Imunodeficiência Adquirida (SIDA) que internarem na UTI a partir de Janeiro/2012. O grupo intervenção receberá terapia ARV entre 2º e 5º dias de internação na UTI e o grupo controle receberá terapia ARV após alta da UTI, de acordo com a equipe assistente. O desfecho primário a ser analisado será mortalidade hospitalar. Os desfechos secundários serão mortalidade em UTI e em 6 meses.

**INTRODUÇÃO E REVISÃO DA LITERATURA**

A terapia anti-retroviral altamente potente e efetiva (HAART, *Highly Active Anti-Retroviral Therapy*) mudou a história natural dos pacientes infectados pelo HIV. Antes do seu advento, a doença comportava-se como potencialmente fatal. Após a introdução de HAART, passou a adquirir características de doença crônica, com melhora na sobrevida [1,2,3]. Apesar dessa melhora, o número de internações em UTI de pacientes infectados pelo HIV permaneceu semelhante nos períodos pré e pós-HAART [4], possivelmente devido ao aumento na prevalência das doenças crônicas nesse pacientes [5,6]. A insuficiência respiratória aguda (IRpA) é o diagnóstico sindrômico mais frequente na admissão à UTI, sendo responsável por até 42% das internações [7]. Dentre as causas de IRpA, pneumonia bacteriana é a principal, seguida pela pneumonia por *Pneumocystis jirovecii*. Com relação ao diagnóstico etiológico, mais da metade das admissões está relacionada a doenças não oportunistas, como co-infecção com vírus da hepatite C e da hepatite B, doença pulmonar obstrutiva crônica, insuficiência renal, cirrose, cardiomiopatias e toxicidade por drogas [8-12].

O prognóstico de pacientes infectados pelo HIV admitidos na UTI, a curto prazo, é determinado pela gravidade da doença que motivou a internação [13,14]. Ainda, quando se compara a mortalidade na UTI pré e pós-HAART não se detecta diferença [4,15]. Com relação à mortalidade a longo prazo, um estudo verificou que os dois fatores de risco independentes associados à mortalidade foram uso de HAART (OR 0,45) e diagnóstico de Síndrome da Imunodeficiência Adquirida (SIDA) na admissão à UTI (OR 2,46) [4].

Em relação ao uso de anti-retroviral, há evidências suficientes para afirmar que infecção pelo HIV não tratada traz consequências negativas à saúde em todos os estágios da doença [16]. Ainda, as novas combinações de drogas anti-retrovirais são mais bem toleradas que regimes prévios, levando a maior eficácia do tratamento e melhora na sobrevida [16]. A recomendação norte-americana, publicada em janeiro de 2011, desenvolvida pelo *Department of Health and Human Services* (DHHS) definiu que o tratamento anti-retroviral deve ser iniciado em todos os pacientes com história de doença definidora de SIDA ou contagem de CD4 < 350 cél/mm^3^ [16]. Dois estudos randomizados que compararam início de ARV com contagem de células CD4 < 350 cel/mm^3^ ou < 200 cél/mm^3^ evidenciaram redução de infecção oportunista e de mortalidade no grupo que iniciou tratamento mais precoce [17, 18]. Além disso, existe uma tendência de tratamento ainda mais precoce (CD4 < 500 cél/mm^3^) devido à evidência que a infecção pelo HIV, por si só, pode contribuir para a maior incidência e progressão de doenças cardiovascular, renal, hepática e neoplasias [19]. Quanto ao início do tratamento durante a internação na UTI, os estudos são escassos. Um estudo retrospectivo em pacientes admitidos na UTI por pneumonia por *Pneumocistis jirovecci* evidenciou diminuição da mortalidade entre os pacientes que já usavam ARV ou que iniciaram durante a internação [20]. Croda et al, em um estudo recente retrospectivo, verificaram que o uso de ARV durante a hospitalização na UTI associou-se a aumento da sobrevida em 6 meses [21]. Outros estudos, no entanto, não conseguiram mostrar diferença na mortalidade na UTI e hospitalar entre os pacientes com e sem HAART [3,4]. Além disso, o uso de ARV em pacientes críticos pode estar associado a graves efeitos colaterais e a aumento de resistência, este último devido à absorção gastrointestinal incerta nestes pacientes. Por fim, a metabolização hepática de muitos ARV pode interferir com o metabolismo de outras medicações administradas na UTI [22].

Com o presente estudo, pretendemos avaliar se o início precoce de ARV em pacientes com infecção pelo HIV admitidos na UTI pode estar associado à redução de mortalidade.

**OBJETIVOS**

**Objetivo geral:** Verificar se há redução de mortalidade hospitalar com início precoce de ARV em pacientes infectados pelo HIV admitidos na UTI.

**Objetivos específicos:** Verificar se há redução de mortalidade na UTI e em seis meses e de tempo de internação na UTI e hospitalar com início precoce de ARV.

**JUSTIFICATIVA**

Não há estudos prospectivos avaliando o uso de ARV na UTI. A partir dos resultados de estudos retrospectivos gerou-se uma hipótese que este uso poderia estar associado à redução de mortalidade. Esta decisão permanece difícil, sendo atualmente baseada na opinião de especialistas.

O presente estudo, por seu desenho prospectivo, pretende trazer significativas contribuições com relação ao impacto do uso de HAART na UTI, avaliando associação com mortalidade, tempo de internação e incidência de infecções oportunistas, além de critérios de segurança.

**MÉTODOS**

**Local de realização:** Unidade de Terapia Intensiva do Hospital Nossa Senhora da Conceição

**Delineamento do Estudo:**

Tipo de estudo: Ensaio clínico

Alocação: Randomização

Cegamento: não cegado

**População:** Pacientes criticamente enfermos admitidos na UTI do HNSC com diagnóstico de SIDA, sem tratamento anti-retroviral prévio, ou sem tratamento regular nos últimos 3 meses antes da internação.

**Período:** Início em janeiro de 2012

**Randomização:** a randomização será feita através de tabela de números aleatórios, em blocos e com estratificação prévia utilizando o escore de gravidade SAPS 3 [23].

**Tamanho da amostra:** Amostra foi calculada com objetivo de redução da mortalidade hospitalar de 15% (sendo a mortalidade no grupo controle 55%), com nível de significância 5% e poder do estudo 80%, necessitando 172 pacientes em cada grupo.

**Grupos do estudo**

**- Grupo Intervenção Precoce:** Pacientes HIV/SIDA, alocados aleatoriamente, para receber ARV entre 2º - 5º dias de internação na UTI

- **Grupo Tratamento convencional:** Pacientes HIV/SIDA, alocados aleatoriamente, para receber ARV após alta da UTI, a critério da equipe assistente.

**Critérios de Inclusão:**

1. Maiores 18 anos

2. HIV positivo

3. a) Contagens de células CD4+ menores ou igual a 350cel/mm^3^ até 3 meses prévio à entrada do estudo

ou

b) Contagem de células CD4+ entre 350cels/mm^3^ e 500cel/mm^3^ se idade superior a 55 anos, coinfecção HCV (vírus da hepatite C) ou HBV (vírus da hepatite B), neoplasia, carga viral >100.000 cópias/ml ou risco cardiovascular elevado (escore de Framingham > 20).

ou

c) Doença definidora de SIDA

**Critérios de Exclusão:**

1. Tratamento regular com HAART

2. Gravidez

3. Meningite tuberculosa

4. Meningite criptocócica

5. Sem condições de utilizar o trato gastrointestinal

6. Permanência na UTI > 5 dias antes da randomização

7. Recusa em participar do estudo

**Critérios de interrupção da intervenção**: Suspensão do tratamento por efeitos colaterais será decidida individualmente pelo infectologista responsável pelo caso. Interrupção do estudo será feita se demonstração de benefício em duas anaálise consecutivas com diferença de quatro desvios-padrão (p unicaudal ≤ 0,00003) durante a primeira metade do estudo  e três desvios-padrão (p unicaudal ≤ 0,002) na segunda metade.

**Coleta de dados:** os dados a serem coletados estão descritos na ficha de coleta de dados (Anexo 1).

**Análise Estatística:** As variáveis contínuas serão apresentadas na forma de média e desvio padrão ou de mediana e intervalo interquartil e comparadas com o teste *t*-Student ou o teste U de Mann-Whitney conforme indicado. As variáveis selecionadas nas análises univariadas (p<0,20) e as variáveis com plausibilidade biológica serão submetidas a análises multivariadas por regressão logística binária. Os resultados das análises uni e multivariadas das regressões logísticas serão expressos em razões de chance e respectivos intervalos de confiança de 95%. Em todas as análises, será adotado como nível para significância estatística um valor de p <0,05.

**CONSIDERAÇÕES ÉTICAS**

A pesquisa observará as recomendações da Resolução n^0^ 196 de 10/10/96 - Conselho Nacional de Saúde (CNS) para Pesquisa Científica em Seres Humanos. Os investigadores comprometem-se em zelar pela *beneficência*, comprometendo-se com o máximo de benefícios e o mínimo de danos e riscos, e pela *não maleficência,* garantindo que danos previsíveis serão evitados. O nosso estudo envolverá introdução de medicações anti-retrovirais em pacientes infectados pelo HIV internados na UTI do HNSC que possuírem critérios de início dessas drogas, fundamentados pelo Consenso Brasileiro de Terapia Anti-retroviral de 2008 e Recomendação Norte-Americana de 2011 [16,24]. O estudo obedecerá à metodologia adequada, com distribuição aleatória dos sujeitos da pesquisa nos grupos de uso de intervenção precoce e tratamento convencional. Além disso, todos os participantes ou responsáveis legais dos mesmos deverão assinar termo de consentimento. Asseguramos que os procedimentos propostos respeitarão a confidencialidade e a privacidade, a proteção da imagem e a não estigmatização, garantindo a não utilização das informações em prejuízo dos pacientes do estudo, inclusive em termos de auto-estima, de prestígio e/ou econômico–financeiro.

Concomitante a isso, garantimos o retorno dos benefícios obtidos através da nossa pesquisa para as pessoas e para a comunidade através de publicações científicas. Os dados serão utilizados apenas para esta pesquisa, serão armazenados por um período de cinco anos e, após, destruídos.

**ORÇAMENTO**

Os materiais necessários à pesquisa, bem como sua quantidade, valor unitário e total estão apresentados na tabela.

Não há previsão de gastos com exames laboratoriais e/ou medicações. Não serão solicitados exames laboratoriais nem será feita prescrição de medicação fora da prática clínica diária. Os gastos previstos serão com material de escritório.

**Orçamento do projeto.**

| **Material** | **Valor unitário (R$)** | **Estimativa total** | **Valor total (R$)** |
| --- | --- | --- | --- |
| Folhas A4 | 0,05 | 2000 | 100,00 |
| Cartucho impressora | 40,00 | 1 | 40,00 |
| Canetas | 2,50 | 4 | 10,00 |
| Total |  |  | 150,00 |

Serão utilizados recursos próprios, sem ônus à Instituição.

**Cronograma**

|  | **2011** | **2012** | **2013** | **2014** | |
| --- | --- | --- | --- | --- | --- |
| **Atividade** / M**ês** | Out a Dez | Jan a Dez | Jan a Dez | Jan | Fev |
| Submissão ao CEP | **X** |  |  |  |  |
| Inclusão de pacientes |  | **X** | **X** |  |  |
| Análise dos dados |  |  |  | **X** |  |
| Elaboração e envio de artigos científicos |  |  |  |  | **X** |

**REFERÊNCIAS**

1. Narasimhan M, Posner AJ, DePalo VA, Mayo PH, Rosen MJ. Intensive care in patients with HIV infection in the era of highly active antiretroviral therapy. *Chest*. 2004;125:1800-1804.

2. Rosen MJ. Intensive care of patients with human immunodeficiency virus infection: Time to take another look. *J Intensive Care Med*. 2005;20:312-313,314,315.

3. Dickson SJ, Batson S, Copas AJ, Edwards SG, Singer M, Miller RF. Survival of HIVinfected patients in the intensive care unit in the era of highly active antiretroviral therapy. *Thorax*. 2007;62:964-968.

4.Casalino E, Wolff M, Ravaud P, Choquet C, Bruneel F, Regnier B. Impact of HAART advent on admission patterns and survival in HIV-infected patients admitted to an intensive care unit. AIDS. 2004;18(10):1429-1433.

5. Morris A, Masur H, Huang L. Current issues in critical care of the human immunodeficiency virus-infected patient. Crit Care Med. 2006;34(1):42-49.

6. Vincent B, Timsit JF, Auburtin M, et al. Characteristics and outcomes of HIV-infected patients in the ICU: impact of the highly active antiretroviral treatment era. Intensive Care Med. 2004; 30(5):859-866.

7. Krista Powell, J. Lucian Davis, Alison M. Morris, Amy Chi, Matthew R. Survival for Patients With HIV Admitted to the ICU Continues to Improve in the Current Era of Combination Antiretroviral Therapy. *Chest* 2009;135;11-17

8. Nickas G, Wachter RM. Outcomes of intensive care for patients with human immunodeficiency virus infection. Arch Intern Med. 2000;160(4):541-547.

9. Rosen MJ, Clayton K, Schneider RF, et al. Intensive care of patients with HIV infection: utilization, critical illnesses, and outcomes. Pulmonary complications of HIV Infection Study Group. Am J Respir Crit Care Med. 1997;155(1):67-71.

10. Afessa B, Green B. Bacterial pneumonia in hospitalized patients with HIV infection: the pulmonary complications, ICU support, and prognostic factors of hospitalized patients with HIV (PIP) study. Chest. 2000;117(4):1017-1022.

11. De Palo VA, Millstein BH, Mayo PH, Salzman SH, Rosen MJ.Outcome of intensive care in patients with HIV infection. Chest.1995;107(2):506-510.

12. Narasimhan M, Posner AJ, DePalo VA, Mayo PH, Rosen MJ.Intensive care in patients with HIV infection in the era of highly active antiretroviral therapy. Chest. 2004;125(5): 1800-1804.

13. Casalino E, Mendoza-Sassi G, Wolff M, et al: Predictors of short-and long-term survival in HIV-infected patients admitted to the ICU.*Chest* 1998; 113:421–429

14. Bhagwanjee S, Muckart DJJ, Jeena PM, et al: Does HIV status influence the outcome of patients admitted to a surgical intensive care unit? A prospective double blind study. *BMJ* 1997; 314:1077–1084

15. Khouli H, Afrasiabi A, Shibli M, Hajal R, Barrett CR, Homel P. Outcome of critically ill human immunodeficiency virus-infectedpatients in the era of highly active antiretroviral therapy. J Intensive Care Med. 2005;20(6):327-333

16. Guidelines for the Use of Antiretroviral Agents in HIV-1-Infected Adults and Adolescents, [http://www.aidsinfo.nih.gov/ContentFiles/AdultandAdolescentGL.pdf](http://../../Library/Mail%20Downloads/%20http:/www.aidsinfo.nih.gov/ContentFiles/AdultandAdolescentGL.pdf), *janeiro, 2011*

17. Zolopa A, Andersen J, Powderly W, et al. Early antiretroviral therapy reduces AIDS progression/death in individuals with acute opportunistic infections: a multicenter randomized strategy trial. PLoS One. 2009;4(5):e5575

18. The SMART/INSIGHT and the D:A:D Study Groups TSIatDADSG. Use of nucleoside reverse transcriptase inhibitors and risk of myocardial infarction in HIV-infected patients. *AIDS.* 2008;22(14):F17-24.

19. Baker JV, Peng G, Rapkin J, et al. CD4+ count and risk of non-AIDS diseases following initial treatment for HIV infection. *AIDS.* 2008;22(7):841-848.

20. Morris A, Wachter RM, Luce J, Turner J, Huang L. Improved survival with highly active antiretroviral therapy in HIVinfectedpatients with severe Pneumocystis carinii pneumonia. AIDS. 2003;17(1):73-80.

21. Croda J, Croda MG, Neves A, De Sousa dos Santos S. Benefit ofantiretroviral therapy on survival of human immunodeficiency virus-infected patients admitted to an intensive care unit. Crit Care Med. 2009;37(5):1605-1611.

22. Huang L, Quartin A, Jones D, Havlir DV. Intensive care of patients with HIV infection. N Engl J Med. 2006;355(2):173-181.

23. Moreno RP, Metnitz PGH, Almeida E, et al: SAPS 3 - From evaluation of the patient to the evaluation of intensive care unit. Part 2: Development of a prognostic model for hospital mortality at ICU admission. *Intensive Care Med* 2005; 31:1345-1355

24. Ministério da Saúde. Recomendações para terapia anti-retroviral em adultos e adolescentes infectados pelo HIV 2007/2008. http://bvsms.saude.gov.br/bvs/ publicacoes/recomendacao_terapia.pdf

**Ficha coleta de dados**

**Admissão na UTI**

Código _____________________

Sexo ( ) Masculino ( ) Feminino

Idade ______

Procedência ( ) Emergência ( ) Enfermaria ( ) Outro hospital

Motivo da internação na UTI___________________________________________

SAPS 3 _______

Internação: No hospital ___/___/___ Na UTI ___/___/___

Tempo de internação prévio à UTI (dias) ___

Diagnóstico de SIDA prévio ( )

Uso prévio de ARVs ( ) Regular ( )

Contagem CD4+ _____ Carga viral _____________

Infecção oportunista na admissão ( ) ___________________________________

Co-Infecções: Hepatite B ( ) Hepatite C ( ) Sífilis ( ) Tuberculose ( )

**Randomização**



Início de TARV na UTI ( )

**Após a randomização**



AZT ( ) 3TC ( ) ddI ( ) d4T ( ) ABC ( ) TDF ( )

EFZ ( ) NVP ( )

LPV ( ) ATV ( ) IDV ( ) NFV ( ) RTV ( ) SQV ( )

APV ( ) ( )_________________________________________________

Data de Início TARV ___/___/___ Dias até início TARV ______

Atraso no início ( ) __________________________________________________

Infecções na UTI ( ) ____________________________________

Infecções oportunistas na UTI ( ) ____________________________________

Efeitos adversos ( ) _______________________________________________

Suspensão ( ) ____________________ Troca ( ) _____________________

Reinício ( ) ___/___/___

Tempo de VM (dias) ____

Data da alta da UTI ___/___/___ Vivo ( ) Dias de UTI _____________

Data da alta hospitalar ___/___/___ Vivo ( ) Dias de hospitalização _____

Vivo seis meses após a alta ( )
