# Supplementary material for: Early antiretroviral therapy for HIV-infected patients admitted to an intensive care unit (EARTH-ICU): A randomized clinical trial
Source: PLoS One. 2020 Sep 21;15(9):e0239452. doi: 10.1371/journal.pone.0239452 (PMC7505451; doi:10.1371/journal.pone.0239452)
Supplement: Attachment — Submitted filename: Response to reviewers.docx [file pone.0239452.s008.docx]

Dear editor,

We would like to thank you and the reviewers for the attention given and the time taken to provide suggestions and corrections to our original study entitled “Early antiretroviral therapy for HIV-infected patients admitted to an intensive care unit (EARTH-ICU): a randomized clinical trial”. We have revised the issues brought up by the editor and reviewer, the point-by-point response can be found below. The changes in the manuscript are highlighted in red.

Please provide additional details regarding participant consent. In the ethics statement in the Methods and online submission information, please ensure that you have specified whether consent was written or verbal/oral. If consent was verbal/oral, please specify: 1) whether the ethics committee approved the verbal/oral consent procedure, 2) why written consent could not be obtained, and 3) how verbal/oral consent was recorded. If your study included minors, please state whether you obtained consent from parents or guardians in these cases.

The informed consent was written. We made this clearer in the manuscript.

We note that you have indicated that data from this study are available upon request. PLOS only allows data to be available upon request if there are legal or ethical restrictions on sharing data publicly. For information on unacceptable data access restrictions, please see [http://journals.plos.org/plosone/s/data-availability#loc-unacceptable-data-access-restrictions](http://journals.plos.org/plosone/s/data-availability#loc-unacceptable-data-access-restrictions).

In your revised cover letter, please address the following prompts:

a) If there are ethical or legal restrictions on sharing a de-identified data set, please explain them in detail (e.g., data contain potentially identifying or sensitive patient information) and who has imposed them (e.g., an ethics committee). Please also provide contact information for a data access committee, ethics committee, or other institutional body to which data requests may be sent.

b) If there are no restrictions, please upload the minimal anonymized data set necessary to replicate your study findings as either Supporting Information files or to a stable, public repository and provide us with the relevant URLs, DOIs, or accession numbers. Please see <http://www.bmj.com/content/340/bmj.c181.long> for guidelines on how to de-identify and prepare clinical data for publication. For a list of acceptable repositories, please see [http://journals.plos.org/plosone/s/data-availability#loc-recommended-repositories](http://journals.plos.org/plosone/s/data-availability#loc-recommended-repositories).

We will update your Data Availability statement on your behalf to reflect the information you provide.

We made the manuscript data available through file upload (as Supporting Information file).

Reviewer #1: This is a well-written study describing the results of a clinical trial to compare outcomes in ICU patients with differing onsets of ART initiation. The study addresses the data from a meta-analysis (Andrade, 2017) on the topic that indicated benefit for initiation of ART in the ICU. The study protocol is well described but recruitment to reach the calculated sample size of 172 patients per group was prematurely ended due to low enrollment with only 115 patients randomized. The failure to enroll the requisite number of patients compromises the trial design and this should be clearly stated in the abstract and throughout the manuscript. The authors need to further explain their rationale for early termination of the trial.

We appreciate the reviewer's comments. We made it clearer in the abstract and in the manuscript that the premature interruption of the trial may have compromised the results.

Minor comments:

Page 4, line 4 – the meaning of “inability to use the gastrointestinal tract” should be clarified.

We replaced "inability to use the gastrointestinal tract" by impossibility to use the enteral route

Page 8 - the meaning of “…patients admitted for AIDS-defining illnesses and with poor immunoblot status…”

We replaced "…patients admitted for AIDS-defining illnesses and with poor immunoblot status…" by "patients admitted for AIDS-defining illnesses and with low CD4+ cell count"

Reviewer #2: Overall, the methods and analyses seem to be performed well. I had a few comments with the aim of improving the description or methods.

1. (p.5) The language here suggests the trial was stopped early because of futility, but on p.6 the says the trip was stopped early due to slow recruitment. I suggest clearing this up and making sure these statements correspond with each other. Also, if there was some sort of planned interim analysis, please describe the criteria that were a priori chosen to stop for futility.

We made it clearer that the trial interruption was due to a progressively slower recruitment rate. We did not perform interim analysis.

2. (p.6) Your variable selection appears to be a combination of bivariate screening and stepwise variable selection. With the sample sizes you have, stepwise variable selection procedures usually do not do a good job of finding the most appropriate model (e.g., <https://doi.org/10.1002/sim.3943>). Stepwise procedures and any p-value based selection have quite a bit of evidence suggesting that they are poor at selecting the appropriate variables. For a decent summary, see the link above. Bivariate screening is really just a form of stepwise selection. Sun et al. (<http://dx.doi.org/10.1016/0895-4356(96)00025-X>) found that bivariate screening can miss a variable that may be a confounder even when a p-value is as high as what you have. Generally, it's better to select based on more robust criteria, especially measures which assess the fit of the model or, better yet, a shrinkage-based estimator such as lasso or lars. I don't know where in SPSS this can be fit, but in R the glmnet package can do it.

The selection of variables for the initial model was based on a combination of biological plausibility and bivariate screening. The regression model was then constructed through stepwise forward. We agree with the reviewer that stepwise forward selection may not be the most appropriate. We applied LASSO as requested by the reviewer. The R script is below:

library(glmnet)

library(ggplot2)

\# Modelo LASSO Hospital Mortality

data_EARTHICU <- read.csv("~/Downloads/data_EARTHICU.csv", sep=";")

data_EARTHICU

xfactors <- model.matrix(data_EARTHICU$Hospital_mortality ~ data_EARTHICU$age + data_EARTHICU$control_treatment_group + data_EARTHICU$origin_ward + data_EARTHICU$AIDSrelated + data_EARTHICU$tuberculosis + data_EARTHICU$pneumocystosis + data_EARTHICU$cryptococosis + data_EARTHICU$saps3 + data_EARTHICU$cd4adm + data_EARTHICU$sepsis + data_EARTHICU$shock + data_EARTHICU$dialysis + data_EARTHICU$MV_adm + data_EARTHICU$ards)[, -1]

glmmod <- glmnet(xfactors, y=as.factor(data_EARTHICU$Hospital_mortality), alpha=1, family="binomial")

plot(glmmod, xvar="lambda")

coef(glmmod)[, 14]

\# Modelo LASSO 6-month mortality

data_EARTHICU6 <- read.csv("~/Downloads/data_EARTHICU6.csv", sep=";")

xfactors6 <- model.matrix(data_EARTHICU6$Mortality_6_month ~ data_EARTHICU6$age + data_EARTHICU6$control_treatment_group + data_EARTHICU6$origin_ward + data_EARTHICU6$AIDSrelated + data_EARTHICU6$tuberculosis + data_EARTHICU6$pneumocystosis + data_EARTHICU6$cryptococosis + data_EARTHICU6$saps3 + data_EARTHICU6$cd4adm + data_EARTHICU6$sepsis + data_EARTHICU6$shock + data_EARTHICU6$dialysis + data_EARTHICU6$MV_adm + data_EARTHICU6$ards)[, -1]

glmmod6 <- glmnet(xfactors6, y=as.factor(data_EARTHICU6$Mortality_6_month), alpha=1, family="binomial")

plot(glmmod6, xvar="lambda")

coef(glmmod6)[, 14]

The shrinkage graph for both models are shown below:

The coefficients for hospital mortality suggest the original model presented is OK, with only dyalisis and shock. LASSO regression coefficients are shown below:

> coef(glmmod)[, 14]

(Intercept)	data_EARTHICU$age

-0.475622602	0.006561752

data_EARTHICU$control_treatment_group	data_EARTHICU$origin_ward

0.000000000	0.000000000

data_EARTHICU$AIDSrelated	data_EARTHICU$tuberculosis

0.000000000	0.081332622

data_EARTHICU$pneumocystosis	data_EARTHICU$cryptococosis

0.000000000	0.241028316

data_EARTHICU$saps3	data_EARTHICU$cd4adm

0.000000000	-0.002027852

data_EARTHICU$sepsis	data_EARTHICU$shock

0.190074265	0.884827694

data_EARTHICU$dialysis	data_EARTHICU$MV_adm

0.664720062	0.000000000

data_EARTHICU$ards

0.000000000

The LASSO coefficients for 6 mo mortality suggest that cryptococcosis should also remain in the final model with dyalisis, shock and tuberculosis. LASSO Coefficients are shown below:

> coef(glmmod6)[, 14]

(Intercept)	data_EARTHICU6$age

-0.15380567	0.00000000

data_EARTHICU6$control_treatment_group	data_EARTHICU6$origin_ward

0.06275091	0.00000000

data_EARTHICU6$AIDSrelated	data_EARTHICU6$tuberculosis

0.00000000	0.71715198

data_EARTHICU6$pneumocystosis	data_EARTHICU6$cryptococosis

0.00000000	0.64094844

data_EARTHICU6$saps3	data_EARTHICU6$cd4adm

0.00000000	0.00000000

data_EARTHICU6$sepsis	data_EARTHICU6$shock

0.00000000	0.69197132

data_EARTHICU6$dialysis	data_EARTHICU6$MV_adm

0.98541680	0.00000000

data_EARTHICU6$ards

0.00000000

In the original logistic regression model presented, cryptococcosis bivariate OR was uncomputable and was removed from the final model due to numerical instability as this variable had an infinite estimated coefficient. This analysis will be available in supplementary material.

3. (p.6) Although I know this is used quite a bit, I don't understand the term "independent predictors" in a multivariable model since the effect is dependent on the other predictors in the model. I recommend something along the lines of "after controlling for other factors". Also, I don't think it's "after multivariate analysis" makes sense since you are pulling the information from the model. Maybe "in the multivariate analysis".

We agreed with the reviewer and made the changes as suggested.

4. It felt like survival analysis might have been a useful analysis in this trial. I'm curious if this was considered. I'm not sure the results would change, but it would seem the timing of the outcomes could have an impact.

We added the survival analysis as Supplemental File.

exp(coef) exp(-coef) lower .95 upper .95

arv$control_treatment_group	0.8591	1.1641	0.5250	1.406

5. Although the tables are pretty standard, I feel as though they need more descriptive titles. For instance, table 2 should say what the numbers are and what tests are used for the p-values. Probably some info on the sample as well. Table 3 should mention the statistical analyses used.

We added the information in the tables as suggested by the reviewer.
